# An Elevated Percentage of Reticulated Platelet Is Associated With Increased Mortality in Septic Shock Patients

**DOI:** 10.1097/MD.0000000000000814

**Published:** 2015-05-21

**Authors:** Qin Wu, Jianan Ren, Dong Hu, Pengjun Jiang, Guanwei Li, Nadeem Anjum, Gefei Wang, Guosheng Gu, Jun Chen, Xiuwen Wu, Song Liu, Yuan Li, Yunzhao Zhao, Jieshou Li

**Affiliations:** From the Department of General Surgery, Jinling Hospital, Medical School of Nanjing University (QW, JR, DH, GL, NA, GW, GG, JC, XW, SL, YL, YZ, JL); and Department of Hematology, First Affiliated Hospital of Nanjing University of Medicine, Nanjing, China (PJ).

## Abstract

Microcirculatory changes and coagulation disturbances are thought to play a key role in sepsis. Some evidence suggests that the percentage of reticulated platelets (RP%) may be a valuable and cost-effective sepsis screening parameter. This was a prospective study in surgical patients to investigate the potential value of RP% as a predictor of mortality in septic shock patients.

This was a prospective study conducted in a surgical critical care center of a Chinese tertiary care hospital. Consecutive septic shock patients were enrolled at admission. Age- and sex-matched non-septic patients were recruited as control patients. RP% was determined by flow cytometry in 68 septic shock patients and 68 controls.

Compared with survivors, septic patients who died presented with a significantly higher RP% (*P* < 0.001). The area under the receiver-operating characteristic curve for the RP% association with mortality was 0.867 (95 % CI 0.780–0.953, *P* < 0.001). Kaplan–Meier survival curves showed that mortality risk was significantly different when patients were stratified based on RP% (*P* < 0.001). This association was preserved in a multi-logistic regression analysis that included clinical confounders (*P* < 0.014).

This prospective study demonstrates that increased RP% identifies septic shock patients who have a high risk of death. RP% has the potential to act as a marker for patient stratification in future clinical trials.

## INTRODUCTION

Sepsis, the systemic inflammatory response to infection, is an aggressive, multifactorial syndrome associated with a high mortality. Despite years of research, sepsis continues to cause high mortality in humans and has a substantial financial impact on health care systems.^1^

In recent years, appropriate treatments for sepsis have demonstrated mortality benefits in general critical care patients.^2,3^ Risk stratification of septic patients (based on major risk scoring systems) is recommended to identify patients who have an elevated risk of death.^4^ The commonly used major risk scoring systems, such as the Acute Physiology and Chronic Health Evaluation scoring system II (APACHE II) score and Sepsis-related Organ Failure Assessment (SOFA) score, identify several important conditions characterizing sepsis. However, these scales have some limitations. For example, the APACHE II score has an exaggerated penalty for old age and does not consider malnutrition or cachexia in the chronic health evaluation^5,6^; prognostic biomarkers are thus urgently needed.

Microcirculatory changes and coagulation disturbances are thought to play key roles in sepsis by activating coagulation factors and platelets.^7^ Therefore, laboratory tests of coagulation markers are widely used to diagnose sepsis and to predict sepsis-induced mortality. Activated platelets, as cells of innate immunity and inflammation, have been demonstrated to play a crucial role in the pathogenesis of sepsis.^8^ Reticulated platelets are immature platelets circulating in the blood. The percentage of reticulated platelets (RP%) is the proportion of reticulated platelets within the total number of platelets. Elevated RP% values have been observed in the setting of acute coronary syndromes and stroke.^9^ Some evidence also suggests that RP% may be useful as a routine laboratory test and as an inexpensive daily screening for bacterial infection in patients with neutrophilia.^10^ In a recent study, RP% was further shown to be useful in predicting the development of sepsis in the emergency department.^11^ However, no study has evaluated the association between RP% and sepsis associated mortality. We therefore conducted a prospective study in trauma and surgical patients to investigate the potential value of RP% as a predictor of mortality in septic shock. Our study indicates that RP% may be able to predict septic shock–related mortality.

## METHODS

### Participants

This was a prospective case–control study conducted in a surgical intensive care unit (SICU) of a tertiary referral hospital in Nanjing, Jiangsu Province, China. The SICU, which belongs to the Department of General Surgery, is primarily used for trauma and post-operative patients. Consecutive patients who had a diagnosis of septic shock at the time of admission to the SICU were enrolled from July 2013 to July 2014. The diagnosis of septic shock was made according to the diagnostic criteria of the American College of Chest Physicians/Society of Critical Care Medicine.^12,13^ An age- and sex-matched population of general surgical critical care patients who had systemic inflammatory response syndrome (SIRS) at the time of admission and who had been treated during the same period were chosen as the control group.

In this study, septic shock was defined as an identifiable site of infection, hypotension that persisted despite fluid resuscitation and required vasopressor therapy, and evidence of a systemic infl ammatory response manifested by at least 2 of the following SIRS criteria: body temperature >38°C or <36°C; heart rate >90 beats/min; respiratory rate >20 breaths/min or PaCO_2_ <4.26 kPa; white blood cell count >12 × 10^9^ cells/L or <4 × 10^9^ cells/L. The site of infection was identified by radiographic testing or bacterial cultures.

The exclusion criteria were as followers: age <18 years; pregnant women; patients with acute coronary disease and stroke; patients with chronic hepatic failure, chronic renal failure, or a known hematologic disease affecting platelets and coagulation; patients with malignancies who were receiving chemotherapy or who had malignancies that affected to the coagulation system or platelets; patients who were taking medications that modified coagulation or platelet behavior; patients who had received platelet transfusions, or fresh-frozen plasma or concentrated blood coagulation products in the previous 1 to 2 days.

All of the patients or their families provided informed consent before participation by their doctors and the study was approved by the ethics committee of Jinling Hospital.

### Blood Sample Collection

To measure the RP%, a venous blood sample was collected from each patient within 2 hours of enrollment and was anti-coagulated with ethylenediaminetetraacetic acid (BD Biosciences, San Jose, CA). To minimize the effect of the anticoagulant on platelets, all measurements were performed within 2 hours of blood collection.

### Flow Cytometry

Flow cytometry analysis was conducted according to previously published procedures.^14,15^ In detail, EDTA anti-coagulated blood samples were centrifuged at 120*g* for 4 minutes. Then the supernatant, which consisted of platelet-rich plasma, was harvested. Ten microliters of supernatant, 20 μL of CD41-PE antibody (BD Biosciences), and 1 mL of thiazole orange (TO, Sigma-Aldrich Shanghai Trading Co Ltd., Shanghai, China) were mixed in the dark for 15 minutes. IgG1-PE (BioLegend, San Diego, CA) was used as the isotype control. In addition, 10 μL of supernatant and 20 μL of CD41-PE antibody without TO were mixed to determine the TO-positive events for each sample. Samples analysis was performed by a Cytomics FC500 cytometer (Beckman-Coulter, Miami, FL). The data were analyzed by using FlowJo software (Treestar, Ashland, OR). The platelet population was gated in an FSC(log)/SSC(log) scatter plot. The selected population was gated to the cytogram and the population of CD41-PE-positive events in the cytogram was gated for this platelet population area. Based on RNA staining by TO, TO positivity was identified by measuring both forward light scatter and green (540 nm) fluorescence using logarithmic amplification among CD41-positive events. The investigator who performed flow cytometry was blinded to the clinical information of the patients. A total of 100,000 events were collected for each sample.

### Data Collection

Clinical and biological variables were collected from each patient at the time of enrollment. The following data were collected: demographic characteristics (age, gender); primary disease (trauma, surgery or others); site of infection (abdominal, pulmonary or others); co-morbidities (chronic obstructive pulmonary disease, chronic heart failure, malignant disease, diabetes, and chronic kidney disease); vital signs (body temperature, heart rate, mean blood pressure); and organ support therapy (mechanical ventilation, renal replacement therapy). Venous blood for all laboratory tests was drawn and was analyzed for the following: C-reactive protein; hemoglobin; platelet counts; international standard ratio; fibrin(-ogen) degradation products; glutamic-pyruvic transaminase; blood urea creatinine; procalcitonin (PCT) and electrolytes. Lactate was also measured in these patients.

Four clinical scores were recorded in our study: APACHE II; the Japanese Association for Acute Medicine disseminated intravascular coagulation (DIC) scoring system (JAAM); the International Society of Thrombosis and Haemostasis (ISTH) score; and the SOFA score when septic shock or SIRS occurred.

Mortality was defined as death occurring within 28 days of admission. We also defined intra-ICU and post-ICU mortality rates; post-ICU mortality was calculated for patients who died while in the hospital but not in the ICU.

### Statistical Analyses

Statistical analyses were performed using SPSS (Statistical Package for Social Sciences, SPSS Inc, Chicago, IL) software for Windows (Version 16.0) and MedCalc software (version 12.0.0.0; MedCalc Software, Mariakerke, Belgium). The values are presented as median and interquartile range (IQR), or mean and standard deviation (SD). A correlation analysis for non-parametric (Spearman's Rho) data was performed to establish relationships between the RP and clinical parameters. The Mann–Whitney test for unpaired data was used for comparisons between the 2 groups. A receiver-operating characteristic curve (ROC) analysis was used to determine the ability of the RP%, the APACHE score, the SOFA score, the PCT level, and the initial lactate level to predict mortality among septic shock patients. The optimal cutoff point was calculated by determining the RP% that provided the greatest sum of sensitivity and specificity. Cumulative survival curves were constructed by the Kaplan–Meier method, and the log-rank test was used to assess significant differences between survival curves. To identify variables that were associated with death, univariate and multivariate logistic regression analyses were performed and odds ratios were estimated with the associated *P* values. *P* < 0.05 was considered significant.

## RESULTS

Eighty-two consecutive septic shock patients were screened for participation in this study. Fourteen of these patients were excluded: 3 for receiving platelet transfusions in the previous 1 to 2 days; 5 for not obtaining informed consent to collect blood samples for measuring RP%; and 6 for delayed blood collection or RP% analysis. The remaining 68 patients were ultimately included in our study. In addition, 68 age- and sex-matched SIRS patients were enrolled as control group. Table 1 displays the demographic data of the study population and Table 2 provides the clinical and laboratory data. All patients in control group survived in 28 days. The APACHE II score, the SOFA score, the JAAM score, the ISTH score, the PCT level, and the initial lactate level were significantly different in the septic shock patients compared with the control patients.

**TABLE 1:**
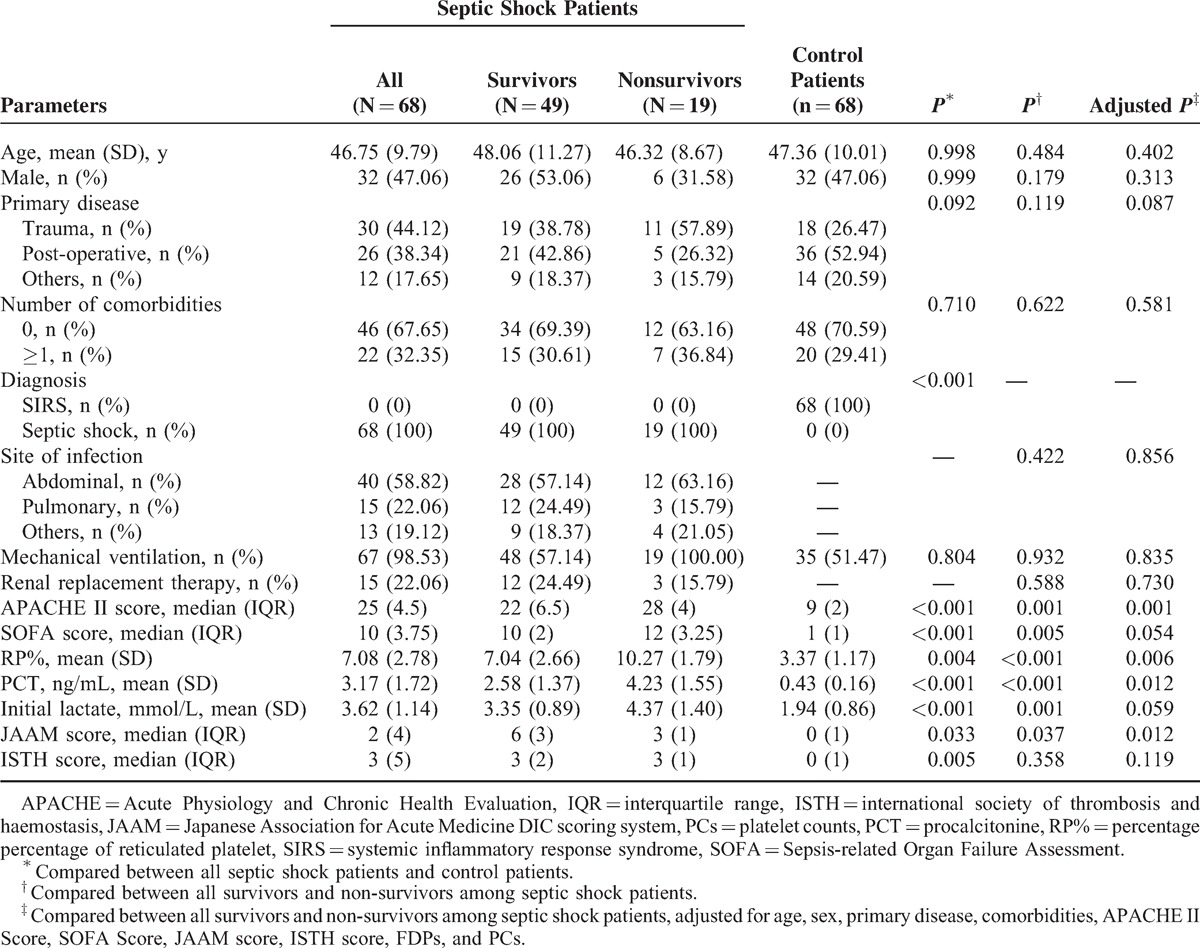
Patient Characteristics for Selected Variables

**TABLE 2:**
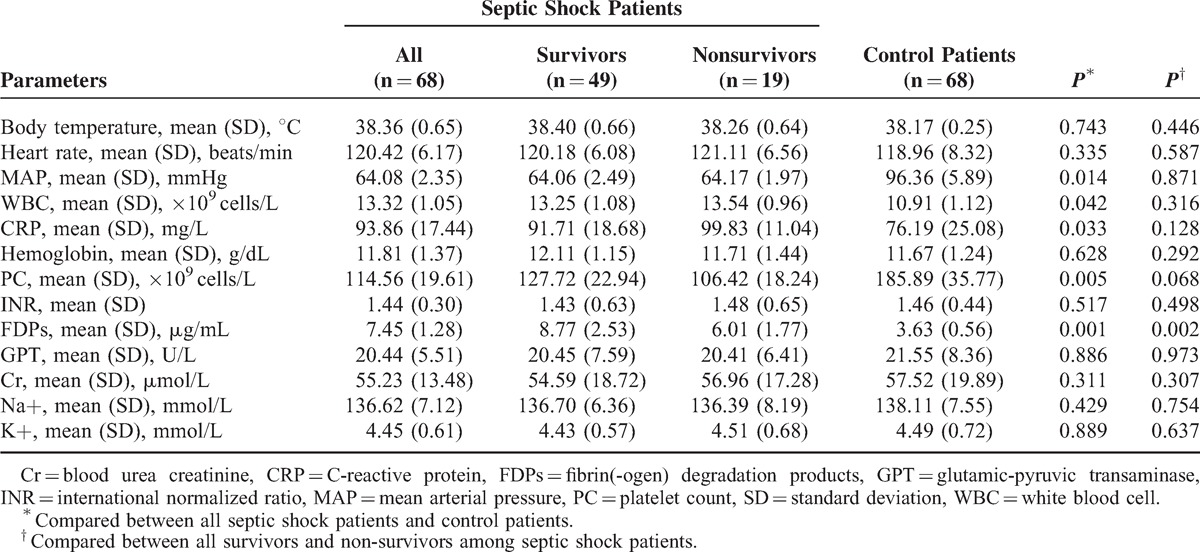
Clinical and Laboratory Data of All Enrolled Patients

We then divided the septic shock patients into 2 groups based on survival at 28 days. No significant differences were observed with regard to regarding age, sex, primary disease, number of comorbidities, site of infection, mechanical ventilation, and the use of renal replacement therapy between survivors and non-survivors (Table 1). A significant difference was observed between these patients in the APACHE II score (*P* = 0.001, adjusted *P* = 0.003), the SOFA score (*P* = 0.005, adjusted *P* = 0.054), the JAAM score (*P* = 0.037, adjusted *P* = 0.012), the PCT level (*P* < 0.001, adjusted *P* = 0.012), and the lactate level (*P* = 0.001, adjusted *P* = 0.059). ISTH score, however, was not significantly different between survivors and non-survivors. Other clinical and laboratory data are listed in Table 2. There were no significant differences between survivors and non-survivors in all indexes examined in our study, except for FDPs (*P* = 0.002).

**FIGURE 1:**
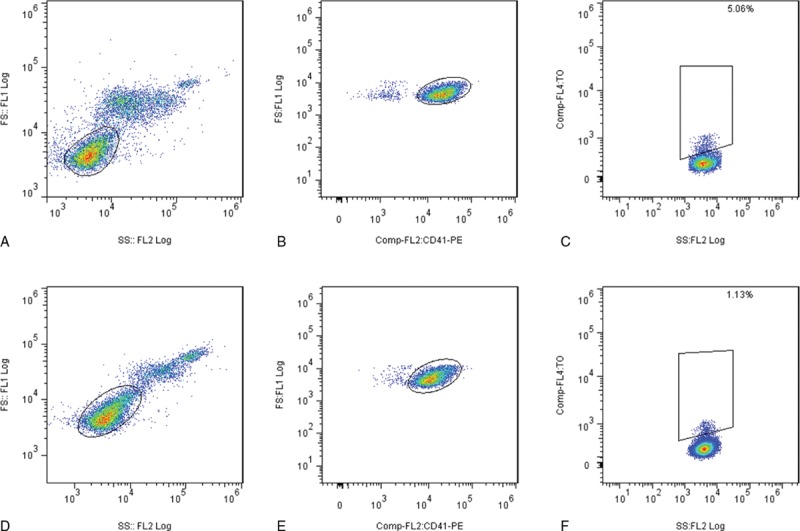
Gating strategies for RP% analysis. Selected patients from the non-surviving group (A, B, and C) and the surviving group (D, E, and F) are shown. Platelets after red blood cell lysis were first plotted in an FSC versus SSC scatter plot (A and D). The platelet population was then plotted in an FSC versus CD41 scatter plot to analyze and precisely determine the platelet population (B and E). The CD41+ population was plotted on the TO/SSC plot (C and F). Irregular gating was demonstrated because of volume-dependent background staining with TO. CD41+TO+ platelets of the appropriate size were viewed as RP. RP% = percentage of reticulated platelets, TO = thiazole orange.

**FIGURE 2:**
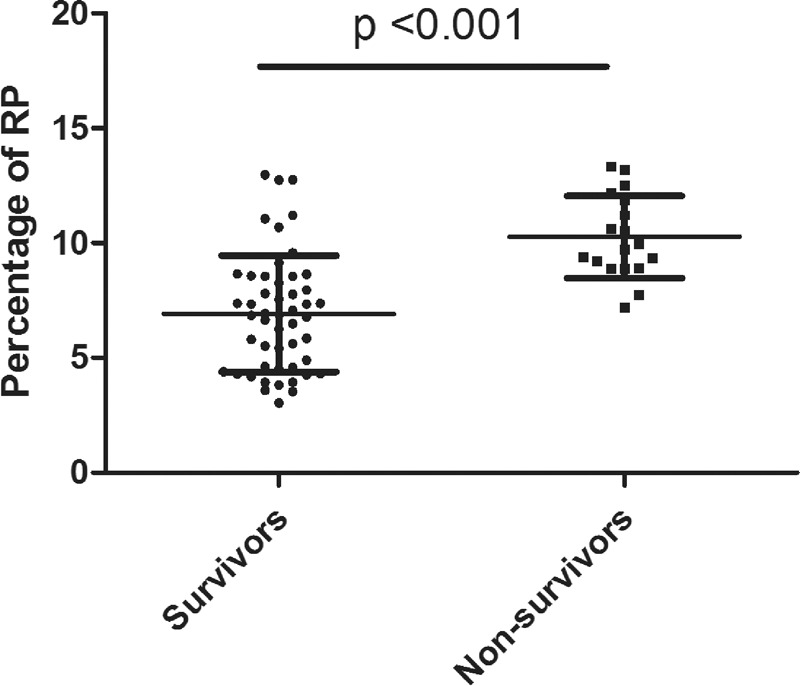
RP% in surviving and non-surviving patients. A significant difference was observed between the groups. Box plot with median and 95% CI. CI = confidence interval, RP% = percentage of reticulated platelets.

RP% was measured in each patient to assess whether it can predict mortality in septic shock patients. The RP% value was significantly higher in septic shock patients who died versus those who survived (*P* < 0.001, adjusted *P* = 0.006, Figure 1, Figure 2, Table 1). Additionally, significant correlations between the RP% and the APACHE II score (*P* < 0.001, *r* = 0.507, Spearman rank correlation coefficients), SOFA score (*P* < 0.001, *r* = 0.471, Spearman rank correlation coefficients), the PCT level (*P* < 0.001, *r* = 0.585, Spearman rank correlation coefficients), and the initial lactate level (*P* < 0.001, *r* = 0.518, Spearman rank correlation coefficients) were observed.

**FIGURE 3:**
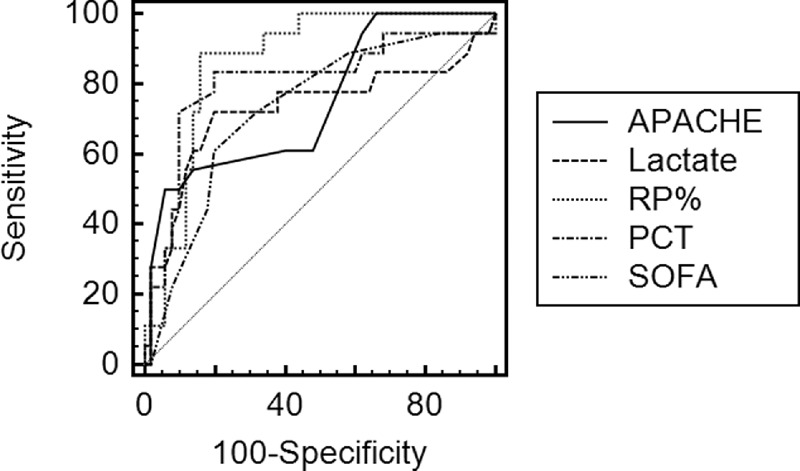
ROC curves of the RP%, the APACHE II score, the SOFA score, the PCT, and the initial lactate level for predicting mortality after septic shock. The areas under the ROC curves for the RP%, the APACHE II score, the SOFA score, the PCT, and the initial lactate level were, respectively, 0.867 (95% CI 0.780–0.953, *P* < 0.001), 0.745 (95% CI 0.610–0.880, *P* = 0.002), 0.729 (95% CI 0.591–0.868, *P* = 0.004), 0.806 (95% CI 0.670–0.941, *P* < 0.001), and 0.730 (95% CI 0.567–0.893, *P* = 0.004). CI = confidence interval, PCT = procalcitonine, ROC = receiver-operating characteristic curve, RP% = percentage of reticulated platelet, SOFA = Sepsis-related Organ Failure Assessment.

The area under the receiver-operating characteristic curve (AUC) for the prediction of 28-day mortality was calculated for the RP%, the APACHE II score, the SOFA score, the PCT level, and the initial lactate level (Figure 3). The AUC for the RP%, the APACHE II and the SOFA score, the PCT level, and the initial lactate level were 0.867 (95% CI 0.780–0.953, *P* < 0.001), 0.745 (95% CI 0.610–0.880, *P* = 0.002), 0.729 (95% CI 0.591–0.868, *P* = 0.004), 0.806 (95 % CI 0.670–0.941, *P* < 0.001), 0.730 (95% CI 0.567–0.893, *P* = 0.004), respectively.

**FIGURE 4:**
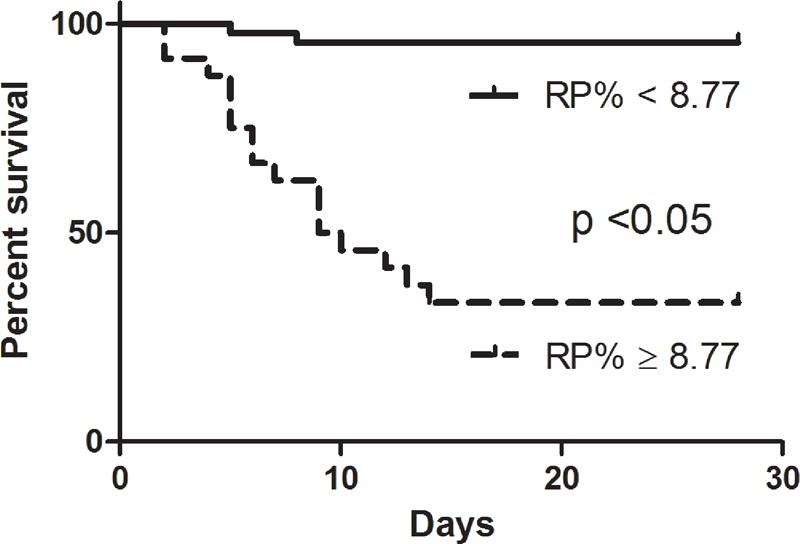
Survival analysis between groups on the basis of an RP% cutoff. Kaplan–Meier survival curves were created based on RP% cutoff values of 8.77%. A significant difference was observed between the 2 curves. RP% = percentage of reticulated platelet.

Kaplan–Meier survival curves were created (Figure 4), using cutoff values that were determined by the ROC curves and based on calculations of the minimum d (RP%, 8.77). The survival rates of septic patients were significantly different when stratified according to the RP%. Patients with lower RP% were more likely to survive than patients with a higher RP% (log rank test, *P* < 0.001). Moreover, as shown in Table 3, patients with a higher RP% had worse clinical outcomes than those with a lower RP% in terms of ICU length of stay (LOS, *P* < 0.001, adjusted *P* = 0.023) and hospital LOS (*P* < 0.001, adjusted *P* = 0.043). Table 4 shows the results of the multivariate Cox proportional hazards analysis comparing patients with high versus low RP%, which indicated that the significant difference in mortality between those 2 groups was not associated with baseline characteristics.

**TABLE 3:**
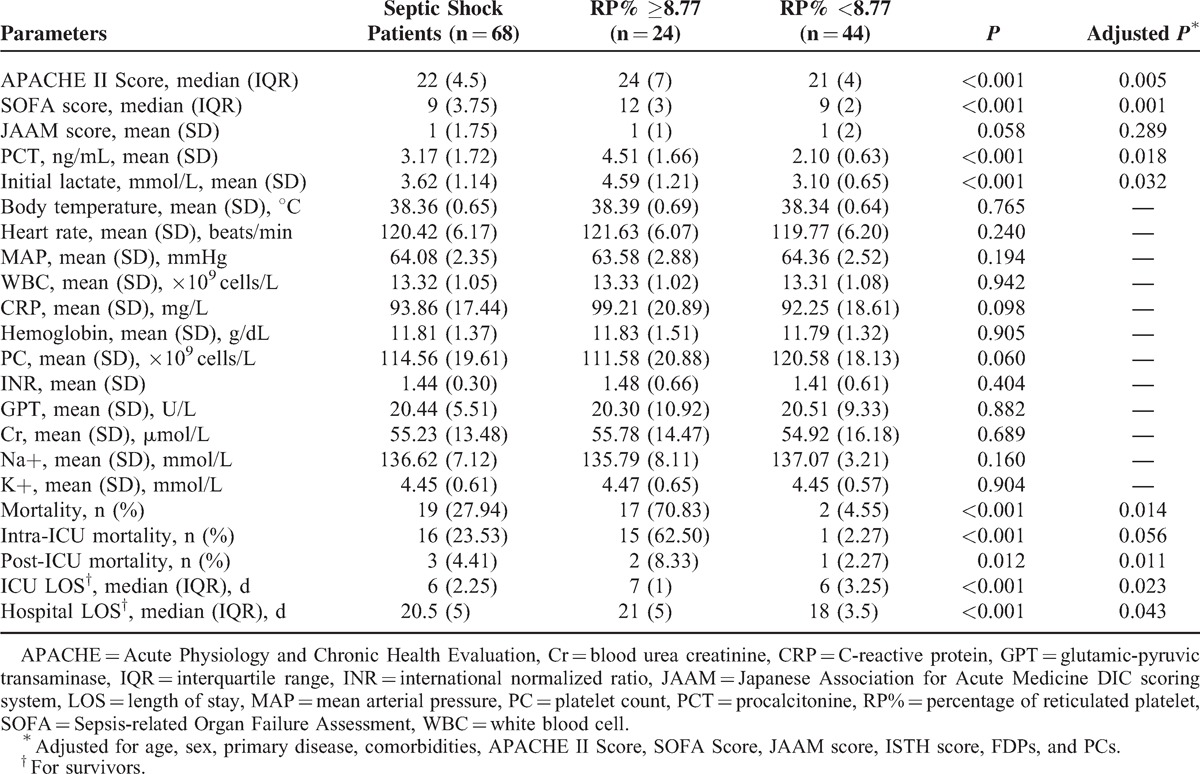
Clinical Data of the Septic Shock Patients Based on RP% Stratification

**TABLE 4:**
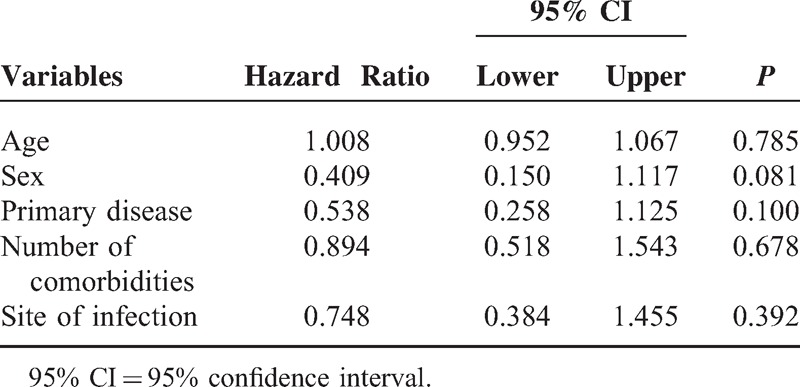
Multivariate Cox Proportional Hazard Analysis

Moreover, using this threshold (RP%, 8.77), the sensitivity of the RP% for differentiating survivors from non-survivors was 88% and its specificity was 84%. The positive predictive value (PPV) was 66%, and the negative predictive value (NPV) was 95%. In comparison, the sensitivity of the APACHE II and the SOFA scores was 50% and 94%, respectively; the specificity was 61% and 80%, respectively. The PPV and NPV for the APACHE II score and the SOFA score were 75% and 83% and 52% and 85%, respectively.

## DISCUSSION

In this study, we prospectively studied septic shock patients to evaluate the potential role of the RP% in predicting septic shock–related mortality. We found that elevated RP% was associated with adverse clinical outcomes, and thus could represent a marker for patient stratification and mortality prediction. Some studies have addressed the subject of sepsis and reticulated platelets^10,16–18^; however, to our knowledge, this is the first study to describe an association between RP% and septic shock mortality.

Despite years of research, sepsis still causes high mortality globally. To help standardize clinical trials and to provide a better understanding of pathophysiology, sepsis has been described as severe sepsis and septic shock. Septic shock is defined as severe sepsis with hypotension and unresponsiveness to fluid resuscitation.^19^ It has been reported that severe sepsis and septic shock are the largest causes of mortality in critically ill patients.^20^ Although the mortality rate of septic shock has decreased in recent years, patient stratification based on biomarkers is still needed to help to reduce sepsis related mortality.^13^

Reticulated platelets are also known as the immature platelet fraction and are a measure of platelet production, thus distinguishing between thrombocytopenia due to increased peripheral platelet destruction and thrombocytopenia related to bone marrow failure caused by toxic agents or a persistent infection.^21,22^ RPs are released early from the bone marrow in response to increased platelet turnover. Blood samples enriched with RPs appear to aggregate more robustly in response to ADP, arachidonate, and collagen and express higher GP II-bIIIa receptor levels compared with older platelets.^23–25^ Moreover, compared with older platelets, RPs more readily express higher levels of surface a-granule factor V, which is necessary for prothrombinase complex formation upon dual agonist stimulation.^26,27^ These combined data suggest that younger RPs are more vigorous than older platelets and that patients with an increased amount of circulating RPs are more susceptible to thrombotic outcomes. Additionally, it has been reported that an early increase in RP% may be a source of procoagulant endotoxins and cytokines in promoting coagulation during sepsis.^28^

Because microvascular changes have an important role in the development of sepsis, the role of RP% in sepsis has been studied. A study conducted by C Scaramucci et al found that RP is a possible predictive index for sepsis.^18^ A similar conclusion was reached by De Blasi et al.^11^ In addition, other studies have supported the notion that RP% is directly associated with the severity of sepsis.^16,17^ In this study, we tesed the hypothesis that RP% is a potential biomarker for identification of patients who are at high risk for death from septic shock. It has been reported that RP% ranges from 0.4% to 6.0% in the healthy population.^29^ The participants in the present study were mainly trauma and post-operative patients. Both trauma and surgery may influence platelet turnover and RP% independent of the presence of sepsis. Thus, we enrolled age- and sex-matched control patients to establish the baseline value of RP%. Compared with SIRS patients, evaluations in the RP% were observed among septic shock patients, indicating the role of coagulation system in sepsis.

We subsequently observed that the RP% levels were associated with increased mortality in a group of septic shock patients. Laboratory data were compared among all participants in whom septic shock occurred; no significant differences between survivors and non-survivors were found, except in FDPs. The association between increased RP% and a higher risk of death persisted in a multivariate analysis that accounted for clinical confounders such as the APACHE II and the SOFA scores. This result suggests that the predictive value of this biomarker is associated with patients’ severity and that this marker may be independently informative. Compared with the APACHE II and the SOFA scores, the results of the ROC analysis indicate the clinical importance of RP%. Based on the threshold determined by the ROC analysis, we further divided our patients into 2 groups and found a significant difference in survival between the 2 groups.

The association between elevated RP% and increased mortality may be multifactorial. The increased RP% can be explained by lower platelet counts in thrombocytopenic patients with or without sepsis. Furthermore, an increased RP% has been correlated with thrombotic events including DIC. In this study, we used the JAAM and the ISTH scores evaluate the role of thrombocytopenia and DIC. We found that the JAAM score was significantly different between survivors and non-survivors, whereas the ISTH score was not. Both the JAAM score and the ISTH score were designed to diagnose DIC. Because the JAAM DIC algorithm is sensitive for the early diagnosis of DIC,^30^ our results indicated that the elevated RP% in this study may be associated with DIC. Additionally, RP% could be a biomarker for DIC before it is manifested in the ISTH score. Moreover, a previous study has suggested that an increased RP% reflects the bone marrow's reaction to sepsis, which occurs before that of the coagulation system.^11^ This result indicates that RP% has considerable potential as a biomarker in sepsis. Because of the role of the coagulation system in the development of sepsis, continuous measurements of RP% may be useful for elaborating the relevant mechanisms.

This study also analyzed 2 established biomarkers in sepsis, lactate, and procalcitonin. Our data demonstrated that RP% was better than procalcitonin and initial lactate for predicting septic shock mortality, which is likely due to the following reasons. Procalcitonin is associated with infection and can discriminate between SIRS and sepsis but may play a limited role in the pathophysiology of shock. Lactate is a good marker for shock; however, in this study, we used initial lactate levels instead of the lactate clearance rate to predict mortality, to ensure comparability among all the analyzed indexes. We therefore may have underestimated the role of lactate in predicting septic shock-related mortality.

The present study demonstrated that an elevated RP% may be associated with the formation of microthrombosis, as discussed above. Thus, RP% may be a potential marker for anti-coagulation therapy in septic shock patients and is a possible stratification parameter in clinical trials. Moreover, the benefit role of recombinant human thrombopoietin as confirmed by our previous study may be due to the stimulation of platelet production and a decrease in the RP%.^31^ However, additional data are required.

Similar to other studies, our study demonstrated that RP% is an index of the severity of sepsis.^16,17^ Our study primarily analyzed the association between RP% and sepsis mortality and indicates that RP% may play an important role in the future. Moreover, our study was larger than previous series.

A number of limitations should be considered when interpreting our findings. Biomarkers provide a snapshot assessment that can be influenced by a variety of factors. In particular, RP%, at least in some cases, may be elevated because of hematologic conditions that are independent of sepsis and its progression. Most of the included patients with septic shock were trauma or post-operative patients, and it is not possible to generalize the present findings to other clinical conditions (eg, medical) that are accompanied by septic shock. Furthermore, in this study, RP% was measured by flow cytometry, which is prone to methodological variations. Many factors contribute to this technical issue, including the type and concentration of fluorescent dyes, the incubation time and temperature, the fixation technique, the use of RNAse treatment, and the selected analytic techniques, including gating and threshold settings; it is therefore difficult to compare the results of different assays.^27^ RP% measurements should be standardized, and cross-validation of results against an automated analyzer would allow more precise measurement of the RP%. This study is solely observational, and the detailed mechanism of the association between increased RP% and mortality is unknown. Additionally, the sensitivity and specificity of the identified RP% threshold as a predictor of septic shock mortality were suboptimal. The 95% confidence intervals of the 3 AUCs are very wide and, in fact, overlap. There are a number of possible explanations for this overlap between the AUCs. Finally, RP% is likely to be only one of a number of variables contributing to the progression of septic shock.

## CONCLUSIONS

In conclusion, our results support the hypothesis that RP% is a novel and attractive way to identify septic shock patients with a higher risk of mortality; RP% is also a potential parameter for patient stratification.
